# Sperm Cohort-Specific Zinc Signature Acquisition and Capacitation-Induced Zinc Flux Regulate Sperm-Oviduct and Sperm-Zona Pellucida Interactions

**DOI:** 10.3390/ijms21062121

**Published:** 2020-03-19

**Authors:** Karl Kerns, Momal Sharif, Michal Zigo, Wei Xu, Lauren E. Hamilton, Miriam Sutovsky, Mark Ellersieck, Erma Z. Drobnis, Nicolai Bovin, Richard Oko, David Miller, Peter Sutovsky

**Affiliations:** 1 Division of Animal Sciences, University of Missouri, Columbia, MO 65211-5300, USA; kkerns@mail.missouri.edu (K.K.); zigom@missouri.edu (M.Z.); hamiltonla@missouri.edu (L.E.H.); sutovskym@missouri.edu (M.S.); ellersieckm@missouri.edu (M.E.); 2 Department of Animal Sciences, University of Illinois at Urbana-Champaign, Urbana, IL 61801, USA; momal.sharif@bcm.edu (M.S.); djmille@illinois.edu (D.M.); 3 Department of Obstetrics and Gynecology, Baylor College of Medicine, Houston, TX 77030, USA; 4 Department of Biomedical and Molecular Sciences, Queen’s University, Kingston, ON K7L 3 N6, Canada; wx@queensu.ca (W.X.); ro3@queensu.ca (R.O.); 5 Department of Obstetrics, Gynecology and Women’s Health, University of Missouri, Columbia, MO 65211-5300, USA; drobnise@health.missouri.edu; 6 Shemyakin Institute of Bioorganic Chemistry, Moscow 117997, Russia; professorbovin@yandex.ru

**Keywords:** sperm, zinc, capacitation, oviductal reservoir, fertilization

## Abstract

Building on our recent discovery of the zinc signature phenomenon present in boar, bull, and human spermatozoa, we have further characterized the role of zinc ions in the spermatozoa’s pathway to fertilization. In boar, the zinc signature differed between the three major boar ejaculate fractions, the initial pre-rich, the sperm-rich, and the post-sperm-rich fraction. These differences set in the sperm ejaculatory sequence establish two major sperm cohorts with marked differences in their sperm capacitation progress. On the subcellular level, we show that the capacitation-induced Zn-ion efflux allows for sperm release from oviductal glycans as analyzed with the oviductal epithelium mimicking glycan binding assay. Sperm zinc efflux also activates zinc-containing enzymes and proteases involved in sperm penetration of the zona pellucida, such as the inner acrosomal membrane matrix metalloproteinase 2 (MMP2). Both MMP2 and the 26S proteasome showed severely reduced activity in the presence of zinc ions, through studies using by gel zymography and the fluorogenic substrates, respectively. In the context of the fertilization-induced oocyte zinc spark and the ensuing oocyte-issued polyspermy-blocking zinc shield, the inhibitory effect of zinc on sperm-borne enzymes may contribute to the fast block of polyspermy. Altogether, our findings establish a new paradigm on the role of zinc ions in sperm function and pave the way for the optimization of animal semen analysis, artificial insemination (AI), and human male-factor infertility diagnostics.

## 1. Introduction

It is widely understood that zinc ions (Zn^2+^) play an important role in male fertility, in species ranging from with *C. elegans* [1] through higher order mammals [2,3,4] (for review, see [5,6,7]), but its role in creating subpopulations of fertilization competent spermatozoa was not known until the discovery of the zinc signature in boar, bull, and human spermatozoa [8]. Although pre-requisite for fertility [9,10], sperm capacitation is a terminal maturation event leading to rapid cell death unless fertilization occurs [11]. The zinc signature is tied directly to key sperm capacitation states: hyperactivation, acrosomal modification, acrosomal exocytosis, and the ability to detect/penetrate the oocyte zona pellucida (ZP) (beginning, midpoint, late, and final capacitation states, [8]), yet the zinc signature states of the oviductal glycan-bound spermatozoa and the role of zinc ions in the sperm release signaling pathways has not been elucidated.

Artificial insemination (AI) is used by a vast amount of the U.S. swine industry (95%+ sows are mated by AI) and is a valuable method to leverage valuable sire genetics and safeguard herd health. Besides the presence of antibiotics in boar semen, currently believed good standard operating procedures discard the first pre-sperm rich fraction due to high bacterial loads [12]. However, literature suggests that spermatozoa in this initial fraction are the most fertile [13], with fewer spermatozoa containing fragmented DNA [14]. Furthermore, spermatozoa from the initial pre-rich fraction are more fit for cryopreservation survival [15] and are overrepresented in the sperm reservoir [16], thus earning its nickname, the “vanguard cohort” [16]. This cohort is much more sensitive to the 26S proteasome inhibition of capacitation than the rich/post-rich fractions [8], indicating a previously unknown, distinct early capacitation event occurring at ejaculation and regulated by sperm-borne proteasomes. What distinguishes these vanguard spermatozoa as biologically different from the rest, remains unknown. Thus, boar semen extenders are not capable of capitalizing on the fertile aspects of this cohort. Additionally, vanguard cohort-friendly semen supplements or extenders have not been developed, which could increase sperm capability to bind to the oviductal sperm reservoir, especially given that sperm processing techniques, such as sex sorting, produce capacitation-like changes that are detrimental to sperm oviductal reservoir binding [17].

Finally, in the molecular/biochemical age, research has lost sight of Chang’s original physiological definition of capacitation [18]: the *acquisition of the capacity to fertilize* [9]. The ability to understand the biology allowing for this fertilization-enabling event could help elucidate male-factor infertility, allowing for the creation of new diagnostic methods that enable physicians to make better recommendations for couples seeking assisted reproductive therapies. It could also enable the creation of media that preserve the spermatozoa’s ability to acquire fertilization competency until the desired time. Similarly, understanding every aspect necessary to acquire this capacity can help positively or negatively control male fertility, as specific applications desire. As we previously proposed, the zinc spark and resulting zinc shield might serve as a new anti-polyspermy defense mechanism [8], and while brain research has suggested the same matrix metalloproteinase-2 (MMP2) co-involved in zona pellucida penetration is inhibited by Zn^2+^ [19], it has not been confirmed whether sperm-borne MMP2 would be susceptible to decreased activity in the presence of Zn^2+^. Likewise, the 26S proteasome, a multi-subunit ubiquitin-dependent protease, regulates multiple steps leading up to fertilization, including sperm capacitation and sperm penetration of the oocyte ZP, as reviewed in [20,21].

## 2. Results

### 2.1. Sperm Zinc Signature is Established by Ejaculatory Sequence

**Figure 1:**
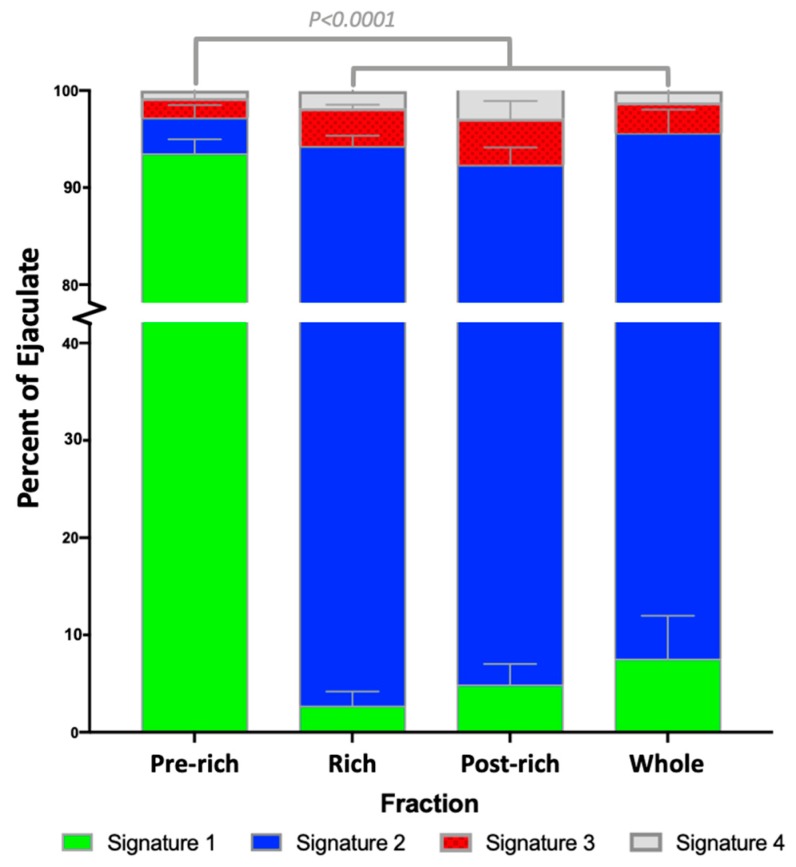
Boar sperm zinc signature differs by ejaculate fraction. Quantitative analysis of the sperm zinc signature across the three ejaculate fractions determined that the pre-sperm rich fraction was significantly different (*p*-value < 0.0001) from the sperm-rich and post-sperm-rich fractions as well as when mixed as a whole ejaculate. Four biological replicates (*n* = 4) were analyzed with a total of 10,000 cells measured for each treatment.

Utilizing high-throughput image-based flow cytometry (IBFC), we examined the sperm zinc signature as reported by fluorescent Zn-tracer FluoZin ™-3 AM (FZ3) across the three main fractions of the boar ejaculate, separated into pre-rich, rich, and post-rich fractions (further defined in the Section 4). We found that the zinc signature is significantly different between ejaculate fractions, with most pre-sperm rich/vanguard group spermatozoa expressing the non-capacitated signature 1 (93.5 ± 1.4%). The rest of the ejaculate possessed mostly signature 2, associated with early stages of capacitation (rich, 91.5 ± 1.1%; post-rich, 87.5 ± 1.8%; whole ejaculate, 88.0 ± 1.8%; Figure 1, summarized in Table 1; *p*-value < 0.0001; *n* = 4 biological replicates; 10,000 spermatozoa analyzed per treatment).

### 2.2. Spermatozoa Posessing Zinc Signatures 1 and 2 Bind Glycans of the Oviductal Sperm Reservior

**Figure 2:**
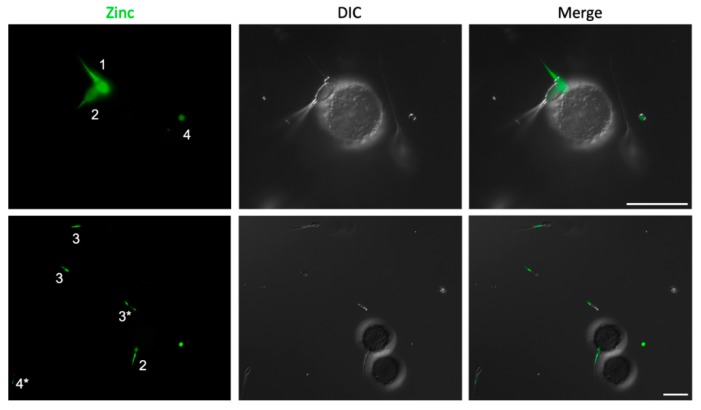
Boar sperm zinc signatures 1 and 2, but not 3 and 4, are capable of binding glycans of the oviductal sperm reservoir. Images in column one are of fluorescent Zn-tracer FZ3 (with zinc signature number in white, asterisk (*) indicates transitioning to enumerated signature); in column two are the differential interference contrast (DIC) images; and in column three are the merged images, representative of findings across three biological replicates. Scale bar: 20 μm.

Glycan-coated streptavidin-sepharose beads linked to biantennary 6-sialylated lactosamine oligosaccharide (bi-SiaLN) and 3-O-sulfated Lewis X trisaccharide (suLe^X^) glycans, which were previously identified as those reciprocal for sperm oviductal binding [22,23], were used to determine the zinc signatures of spermatozoa capable of binding the oviductal sperm reservoir. Using spermatozoa from the entire boar ejaculate (pre-rich, rich, and post-rich), we identified that only those spermatozoa possessing zinc signatures 1 and 2, but not zinc signatures 3 and 4, were bound to glycans mimicking those of the oviductal sperm reservoir (Figure 2).

### 2.3. Externally Added Zinc Ions Inhibit Sperm Release from Glycans of the Oviductal Sperm Reservior

**Figure 3:**
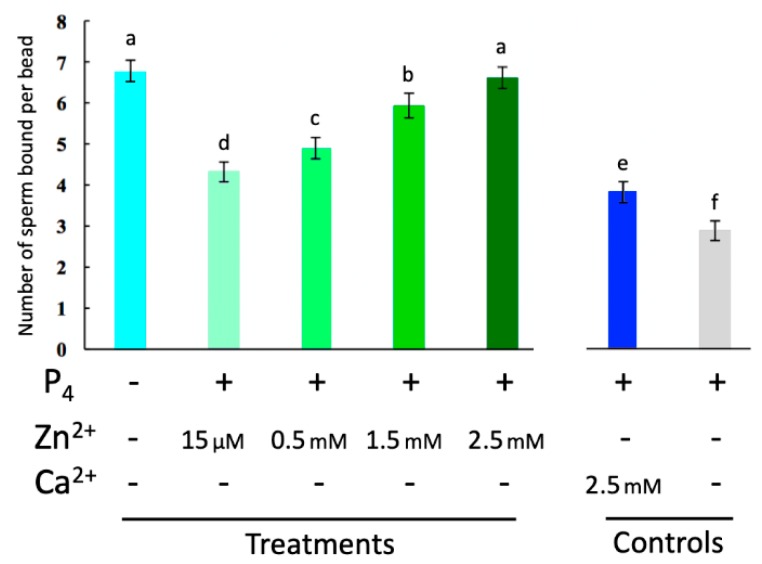
Zinc inhibits P_4_-induced sperm release from oviductal glycans. Quantitative analysis revealed that Zn^2+^ inhibits P_4_-induced sperm release from oviductal glycans in a dose-dependent manner, with 2.5 mM Zn^2+^ being significantly different from the 2.5 mM Ca^2+^ control. Different lowercase letters (a,b,c,d,e) indicate significant difference across treatments (*p*-value < 0.05). Three biological replicates (*n* = 3) were analyzed. Additional controls are shown in Figure S1 and numerical data in Table S1.

While the relative concentration of Zn^2+^ in seminal fluids is the highest of all bodily fluids (boar [24], human [25]), ranging from 2.0–3.0 mM, the concentration of Zn^2+^ decreases as the spermatozoa reaches the site of fertilization to less than 15 μM, a concentration less than that of blood serum [26,27]. We thus set out to examine if this decrease in zinc concentration was necessary for sperm release from glycans of the sperm oviductal reservoir in response to ovulatory signal and sperm-oviduct release cue progesterone (P_4_) [28]. Varying concentrations physiologically representative of those of spermatozoa on the path to the oviductal reservoir were examined. Our oviductal glycan assay showed that all concentrations of Zn^2+^ examined (15 μM, 0.5 mM, 1.5 mM, and 2.5 mM) prevented P_4_-induced release from oviductal glycans (Figure 3; *n* = 3 biological replicates; *p*-value < 0.05). Sperm release was in proportion to the concentration of Zn^2+^, with concentration representing seminal fluids (2.5 mM) completely inhibiting sperm release.

### 2.4. Sperm Head Zn-Efflux Occurs in a Posterior-to-Anterior Modification Wave and Localizes to Sperm Acrosome

**Figure 4:**
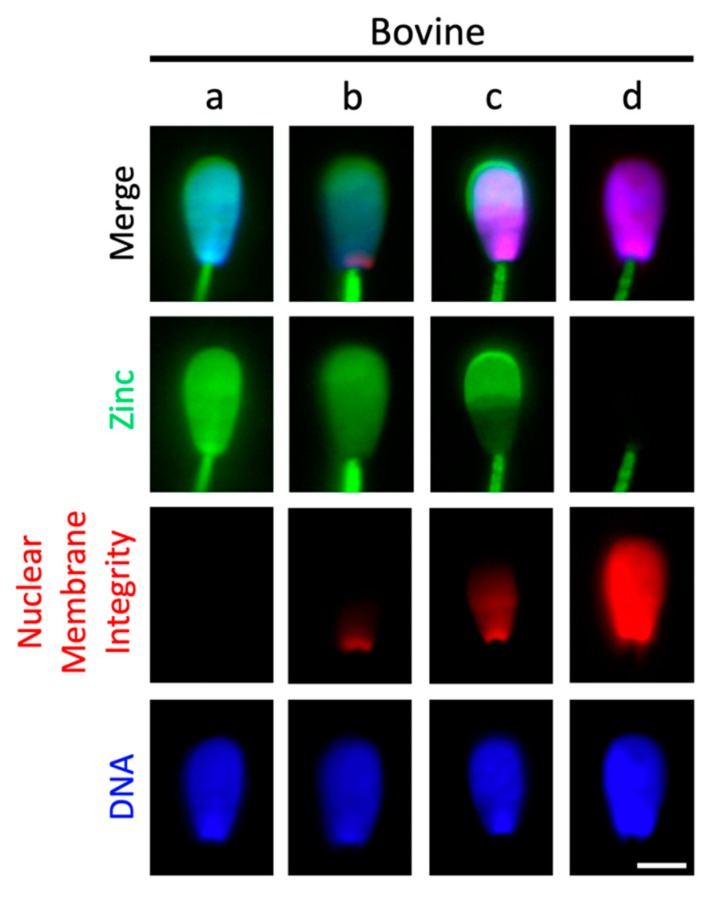
Bull sperm head Zn-efflux, reflected by the transition from signature 2 to 3, is associated with a posterior-to-anterior nuclear membrane modification wave. (**a**) Epifluorescence microscopy of entire sperm-head Zn localization at zinc signature 2 (green). (**b**) Plasma membrane and nuclear envelope modification-reflecting probe propidium iodide (red) showing initial membrane modification originating at the posterior sperm head and lesser Zn-probe fluorescence intensity. (**c**) Increased membrane modification enabled the exodus of Zn-probe fluorescence from the post-acrosomal sheath with remaining fluorescence restricted to the acrosome. (**d**) Full acquisition of sperm zinc signature 3 state, with propidium iodide localized to the entire sperm nucleus and the removal of acrosomal Zn (detectable by lack of FZ3 fluorescence). DNA/Nucleus in blue. Scale bar: 5 μm.

Understanding that the main difference between glycan-bound versus unbound spermatozoa is zinc localized to the sperm head, we set out to describe the transition from zinc signature 2 to zinc signature 3. We observed that this transition occurs in a matter of 2–3 s in fresh boar spermatozoa; thus, we were unable to capture still images. However, this transition is slower (~2–3 min) in frozen-thawed bull spermatozoa and therefore we were able to acquire still images of the sperm head zinc efflux and changes in the integrity of the sperm plasma membrane and nuclear envelope in this species (Figure 4). The head of a signature 2 spermatozoon is shown in Figure 4a, with zinc localized to the entire sperm head and intact membranes, reflected by lack of propidium iodide (PI) labeling. Upon early plasma membrane modification (Figure 4b), PI can be seen infiltrating the posterior aspect of the sperm head while a relative decrease in FZ3 fluorescence is observed in the post-acrosomal sheath. As more PI is incorporated into the sperm head, zinc is reported in the bull sperm acrosome (Figure 4c) until finally, full PI incorporation into the sperm nucleus and no acrosomal localized FZ3 is observed (Figure 4d).

### 2.5. External Zinc Inhibits the Activity of Sperm Proteinases

**Figure 5:**
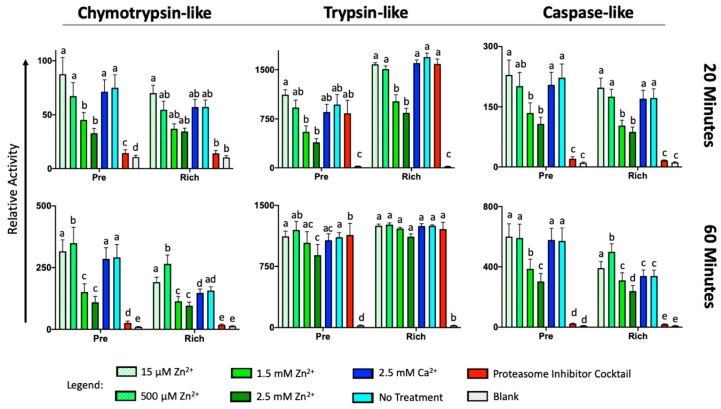
Zinc influences the activity of capacitation-regulating and zona pellucida (ZP)-digesting 26S proteasome in an ejaculate fraction-dependent mechanism. The activity was assessed using fluorogenic substrates Suc-LLVY-AMC (for chymotrypsin-like activity), Ac-RLR-AMC (trypsin-like activity), and Ac-GPLD-AMC (caspase-like activity). The significance between treatment within a fraction of *p* < 0.05 is indicated by superscripts (a,b,c,d,e). Five biological replicates were analyzed, with four technical replicates per treatment/biological replicate. The proteasome inhibitor cocktail used had no significant effect in attenuating trypsin-like activity.

**Figure 6:**
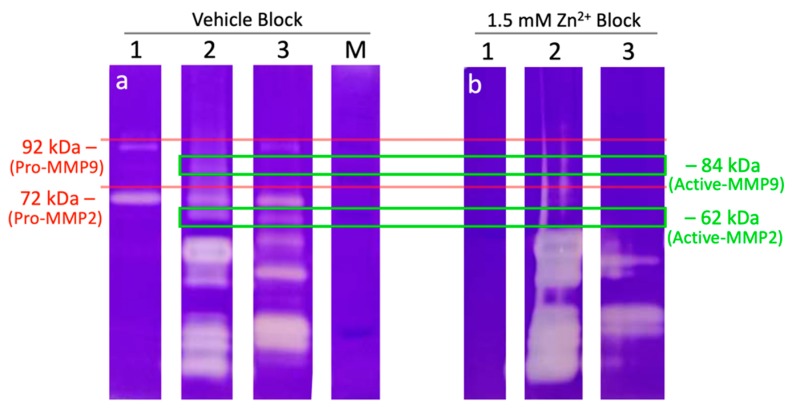
Zinc ions block the activity of sperm-borne ZP proteinase matrix metalloproteinase-2 (MMP2). Clear bands in the zymogram indicate the enzymatic digestion of gelatin. (**a**) Both boar and bull sperm extracts show enzymatic digestion at 72 and 92 kDa in the vehicle block, that of expected pro-MMP2 and MMP9 activities, respectively, as well as 62 kDa expected weight of active-MMP2. (**b**) The same bands show a decreased enzymatic gelatin digestion when blocked with 1.5 mM Zn^2+^ while up-regulating the activity (increased enzymatic digestion) of the lower mass proteinases. Validation of this technique for sperm MMP activity, including MMP inhibitor GM6001, is covered in [33]. Red lines are over pro-MMP bands expected to be affected by treatment and green boxes are over active-MMP forms. Results typical of two biological replicates (*n* = 2) are shown. Lanes: (1) trophoblast cell line expressing pro-MMP2 and MMP9 (positive control); (2) boar sperm extract; (3) bull sperm extract; (M) marker.

Finally, we examined whether the fertilization-induced oocyte zinc spark and resulting zinc shield’s inhibition may serve as a possible anti-polyspermy defense mechanism. While ZP proteinase acrosin has already been shown to be inhibited by Zn^2+^ [29], mouse knockout studies have shown that acrosin may not be the sole ZP-targeting proteinase/zona lysin [30]. Furthermore, sperm proteasomes have been implicated in ZP-penetration in various vertebrate [31] and invertebrate [32] species, as reviewed in [21]. It has been newly discovered that sperm Zn-metalloproteinase MMP2, located on the sperm inner acrosomal membrane (IAM), is an additional sperm-borne zona lysin [33]. We used fluorogenic substrates to examine all three proteolytic activities of the 26S proteasome (chymotrypsin-like, trypsin-like, and caspase-like) in the presence of the same physiologically relevant concentrations of Zn^2+^ used in the oviductal glycan release assay. We found that increased concentrations of Zn^2+^ (1.5 and 2.5 mM) reduced all three sperm-proteasomal activities at 20 min of incubation in both the pre-rich and rich fractions (Figure 5), with the exception of sperm chymotrypsin-like activity of the rich fraction. Interestingly, after 60 min of incubation, 500 µM Zn^2+^ increased the chymotrypsin-like activity of both fractions and the caspase-like activity of spermatozoa from the sperm rich fraction, while no change in activity was observed in caspase-like activity of spermatozoa from the pre-sperm rich fraction (*p* < 0.01, *n* = 5 biological replicates, four technical replicates per treatment/biological replicate). Additionally, to understand if Zn^2+^ blocks sperm-borne zona pellucida proteinase MMP2, we used zymography paired with the Zn^2+^ blocking of boar and bull sperm extracts. The presence of 1.5 mM Zn^2+^ prevented the gelatin breakdown of 72 and 92 kDa proteins (MMP2 and 9, respectively) as well MMP2 and MMP9 expressing trophoblast cell line extract (positive control) while smaller sized proteins had increased gelatin breakdown in boar as compared to vehicle block (Figure 6, *n* = 2 biological replicates). Thus, we can confirm that sperm-borne MMP2 activity is severely reduced in the presence of Zn^2+^ concentrations similar to seminal fluid while upregulating other proteins with gelatinase activity.

## 3. Discussion

A vast majority of sperm function studies rely strictly on a biochemical perspective, at times with little regard for the basic ejaculate physiology. Reflective of a widespread misunderstanding of seminal plasma is the belief that it is a relatively homogenous fluid, like that of blood plasma, a fluid with well-regulated homeostasis. Contrarily, seminal plasma is anything but this, thus referred to as seminal fluids by some [34]. The zinc signature differences uncovered in this report between the vanguard group (pre-sperm rich fraction) and the rest of the ejaculate can likely be attributed to the composition of seminal fluids engulfing the respective fractions. The pre-rich fraction fluid originating from the prostate is known to have the largest amounts of free Zn^2+^, while later portions of the ejaculate containing mostly vesicular gland fluids are low in Zn^2+^, at least in humans [35]. While boar seminal vesicular fluids contain Zn^2+^ [36], most of this free Zn^2+^ is chelated by seminal vesicle citrate [37] and may be bound to other Zn-interacting seminal fluid proteins, such as spermadhesin PSP-1 [38]. Additionally, progression through the later ejaculate fluids entails a rise in pH in the presence of sperm motility-activating components [39]. Given that vanguard group spermatozoa are more capable of binding the oviductal reservoir than the remainder of the ejaculate [16], combined with the knowledge that only non-capacitated spermatozoa are capable of binding, it comes as no surprise that there is a difference in the capacitation-associated zinc signatures of these fractions. It is also no surprise that spermatozoa with zinc signatures 1 (non-capacitated) and 2 (early capacitation) bind to glycans of the sperm oviductal reservoir, as both of these have been previously reported to have no acrosomal modifications [8]. To the contrary, signature 3 and 4 spermatozoa, representing the late stages of capacitation and complete capacitation, respectively, display acrosomal membrane modifications permissible to lectin peanut agglutinin (PNA) binding, and even spontaneous (as in not induced by sperm-zona binding) acrosomal exocytosis [8]. Future studies should determine if spermatozoa possessing signature 1 have an increased ability to bind these oviductal glycans in comparison to signature 2 spermatozoa, given that vanguard group spermatozoa have an increased ability to bind the epithelium of the oviductal sperm reservoir [16]. If such is true, together, these zinc signature differences, pre-determined by the ejaculatory sequence, might destine which spermatozoa have an increased binding affinity to glycans of the oviductal epithelium, quiescently awaiting later ovulated oocytes (the pre-sperm rich, zinc signature 1 spermatozoa). The sperm-rich and post-sperm-rich fraction spermatozoa that have undergone the early stages of Zn-signaling capacitation (Signature 2) might be destined as those that are released first upon ovulatory signal (compared to non-capacitated signature 1 spermatozoa) or for immediate fertilizing of ovulated, fully mature oocytes upon the time of mating/insemination. Alternatively, this later sperm cohort and seminal fluids could serve as a vehicle to stimulate uterine and oviductal epithelial cells’ response to semen deposition, priming for imminent embryo implantation. The theory of seminal fluids eliciting a stimulatory response to prime for pregnancy [40] is supported by the meta-analysis of human publications. Meta-analysis confirmed an improvement in in vitro fertilization pregnancy rates when couples had sexual intercourse around the time of oocyte pick-up or embryo transfer [41]. Understanding the zinc signature state of the sperm cohorts in relation to the time of insemination and ovulation might prove useful in the creation of new semen extenders that are specifically formulated for inseminations prior to detectable ovulation and timed-artificial insemination.

It is unclear if the posterior-to-anterior sperm head PI changes observed are indicative of an entire membrane change in a posterior-to-anterior directed wave, or if this is indicative of simply PI crossing the modified (i.e., more fluid and permeable) sperm plasma membrane and nuclear envelope in the post-acrosomal region. However, since Zn^2+^ removal mimics this pattern, it is likely the former. The FZ3 fluorescence localized to the bull acrosome likely represents Zn^2+^ attached to or dissociated from acrosomal Zn-sensing receptor (ZnR) GPR39 of the G-protein-coupled receptor. This ZnR has been reported previously in bull spermatozoa and is believed to regulate acrosomal exocytosis [42]. Additionally, the sperm life span following the posterior-to-anterior sperm head membrane modification was relatively short (less than 5 min) and is presumed to be the time at which full molecular acquisition or competence to fertilize comes as no surprise. Previous studies have suggested reactive oxygen species (ROS) creation is critical to capacitation success [43], yet this is a risky strategy for spermatozoa due to their lack of antioxidant protection. If true, herein lies the origin of current under-appreciation of sperm ion fluxes within sperm fertilization biology.

Still image acquisition of the posterior-to-anterior sperm head membrane modification might have been possible in bull spermatozoa due to the use of cryopreserved spermatozoa (whereas fresh spermatozoa from boar was used). It is known that cryopreservation inflicts freezing-induced, capacitation-like changes on spermatozoa. It is unclear if these changes occurred slower in bull compared to boar because of freezing-induced damage or if it may be due to species differences in sperm plasma membrane composition, such as different content of cholesterol (presumably higher in bull). If this is a side effect of cryopreservation, then cryopreservation could affect sperm Zn-signaling modifications and sperm fertility. Routine assessment of the zinc signature could prove useful in future attempts to improve cryopreservation methods.

While it was previously known that Zn^2+^ serves as a de-capacitating factor by inhibiting hydrogen voltage channel HVCN1 [44], we now show it also serves this function by decreasing the proteolytic activities of the sperm 26S proteasome and MMP2, and by preventing the release from glycans of the oviductal reservoir. This further supports the sperm release from the oviductal reservoir being a sperm capacitation-driven event in response to female reproductive tract ovulatory cues, rather than a female reproductive tract-oviductal glycan degradation-related release, as discussed by others [45]. Zinc has been implicated in inhibiting 26S proteasome-dependent proteolysis in HeLa cells [46] and the 20S proteasomal core activity in bovine brain tissue [47]; however, until now it was unknown if Zn^2+^ would inhibit or reduce sperm 26S proteasome-dependent proteolysis. The increase in all three activities of the 26S proteasome in response to reduced Zn^2+^ concentration is physiologically relevant, increasing these sperm activities as seminal fluids high in Zn^2+^ are washed away from spermatozoa on their journey through the female reproductive tract, especially given the multi-faceted role of the 26S proteasome in regulating sperm capacitation [20]. Furthermore, inhibition of the 26S proteasome has been shown to reduce spermadhesin de-aggregation from the sperm surface, one of the major proteins participating in formation of the oviductal sperm reservoir [48].

The major role of zinc in the sustenance of zinc-containing proteins can be catalytic, cocatalytic, or structural [49]. Zinc is a required ion for many MMP activities, including MMP2 and MMP9. Both MMPs have two Zn^2+^ sites and four (MMP2) and three (MMP9) Ca^2+^ sites (UniProt Acc no.: P08253 and P14780) [50]. A finely tuned balance in zinc:calcium ratio is therefore important for MMP activity [51]. At a high concentration, Zn^2+^ binds competitively the Ca^2+^ site, thereby causing a structural change and inhibiting the proteinase activity of MMPs [19]. Conversely, when the Zn^2+^ ion is chelated, MMP activity is inhibited [52]. Environmental exposure to divalent cation cadmium in rats has shown decreases in both MMP2 and MMP9 testicular tissue activities [53], likely by competitive binding to Zn^2+^ and/or Ca^2+^ sites. We likewise found that zinc inhibited human sperm MMP2 activity (Figure S2). Interestingly, low molecular weight sperm proteinases were upregulated in our Zn^2+^ block (Figure 6) and deserves future exploration. Thus, as we originally proposed that the oocyte-issued zinc shield may serve as an anti-polyspermy defense mechanism [8], we herein prove the precise mechanism—inhibition of two major ZP proteinases, the 26S proteasome and MMP2. Given that Zn^2+^ repels spermatozoa that are susceptible to P_4_ chemotaxis [54], this pathway may offer a new, non-hormonal contraceptive target. This target could be designed for the male or female, as an abundance of Zn^2+^, compounds that competitively target catalytic Zn^2+^ sites, or chelation of Zn^2+^. Chelation of MMP2 bound Zn^2+^ by application of topical carbonic anhydrase inhibitors has served as a biological exploitation in lowering intraocular pressure in glaucoma patients [55].

## 4. Materials and Methods

### 4.1. Reagents

All reagents unless otherwise noted were from Sigma. FluoZin^TM^-3, AM (FZ3; zinc probe) from ThermoFisher (F24195) was reconstituted with DMSO to a stock solution of 500 μM. Hoechst 33342 (H33342) from Calbiochem (382065) was reconstituted with H_2_O to a stock solution of 18 mM. Propidium Iodide (PI) from Acros Organics (AC440300010) was reconstituted with H_2_O to a stock solution of 1 mg mL^−1^. Proteasomal inhibitors were from Enzo Life Sciences: MG132 (BML-PI102) was reconstituted with DMSO to a stock solution of 20 mM; Epoxomicin (Epox, BML-PI127) was reconstituted to a stock solution of 20 mM (using MG132 stock); and clasto-*Lactacystin* β-Lactone (CLBL, BML-PI108) was reconstituted with DMSO to a stock solution of 5 mM. For the proteasome activity assay, the following reagents were purchased from Enzo Life Sciences: adenosine 5’-triphosphate disodium salt (ALX-480-021), Ac-GPLD-AMC, (BML-AW9560), Ac-RLR-AMC (BML-AW9785), and Suc-LLVY-AMC (BML-P802-000). Digitonin (300410) was purchased from Millipore Sigma (Burlington, MA, USA).

### 4.2. Models, Semen Collection, and Processing

Domestic boar (*Sus scrofa)* semen collection was performed under the guidance of approved Animal Care and Use protocols of the University of Missouri-Columbia and purchased from the National Swine Research and Resource Center (University of Missouri, Columbia, MO). Boar collection was performed using the standard two gloved hand technique [56]. Only ejaculates with greater than 80% motility were used. Semen was immediately extended, within 2 °C, five times in Beltsville thawing solution (BTS) semen extender unless otherwise specified. Sperm concentration was then determined using a hemocytometer. All washes were performed with a swing hinge rotor centrifuge at 110× *g* for 5 min. The frozen-thawed bull (*Bos taurus*) spermatozoa were processed similarly as boar spermatozoa after being thawed for 45 s in a 35 °C water bath. For human spermatozoa, sperm donors signed informed consent and the samples were coded as to make the donors unidentifiable to researchers. All human sperm samples were handled and processed strictly as stipulated by an approved Internal Review Board (MU IRB) protocol. Donors were recruited by placing an advertisement for new fathers in the university mass e-mail newsletter. All semen was collected onsite at the Missouri Center for Reproductive Medicine and Fertility Clinic. Samples were then transported to the laboratory for analysis.

### 4.3. Collection and Processing of Spermatozoa for Zinc Signature Fraction Analysis

For each replicate, semen was collected from 3 to 5 mature *Sus scrofa* boars and analyzed immediately. The semen was washed in TL-HEPES within 30 min after collection. Only samples with greater than 80% sperm motility were used for experiments. If any fraction was contaminated with urine, the entire replicate was discarded from data analysis. The pre-sperm rich fraction was identified as the first clear fluids, preceding the milkier sperm-rich fraction, and was 5–12 mL. The sperm-rich fraction ranged in 35–50 mL and the post-rich fraction, which was less milky but whiter than the pre-sperm rich fraction, ranged from 70–110 mL.

### 4.4. Multiplex Fluorescence Probing

A sample size of 100 μL and 4 million spermatozoa were incubated for 30 min with 1:200 H33342, 1:200 PI, and 1:100 FZ3 for epifluorescence microscopy at room temperature in TL-HEPES. Lower probe concentrations were necessary for image-based flow cytometry (IBFC) due to camera detection differences, thus 1:1000, 1:1000, and 1:500 were used, respectively. The spermatozoa were then washed of probes once and resuspended to allow complete de-esterification of intracellular AM esters, as suggested by ThermoFisher’s FZ3 protocol, followed by an additional wash and resuspended in 100 μL PBS for IBFC analysis (or added to a slide for epifluorescence microscopy imaging).

### 4.5. Epifluorescence Microscopy Imaging

Live spermatozoa were imaged using a Nikon Eclipse 800 microscope (Nikon Instruments Inc.) with Cool Snap camera (Roper Scientific, Tucson, AZ, USA) and MetaMorph software (Universal Imaging Corp., Downingtown, PA, USA). Images were adjusted for contrast and brightness in Adobe Photoshop 2020 (Adobe Systems, Mountain View, CA, USA) to match the fluorescence intensities viewed through the microscope eyepieces.

### 4.6. Image-Based Flow Cytometric Data Acquisition

IBFC data acquisition was performed following the previous methodology [57]. Specifically, using a FlowSight flow cytometer (FS) fitted with a 20× microscope objective (numerical aperture of 0.9) with an imaging rate of up to 2000 events per sec. The sheath fluid was PBS (without Ca^2+^ or Mg^2+^). The flow-core diameter and speed were 10 μm and 66 mm per second, respectively. The raw image data were acquired using INSPIRE^®^ software (AMNIS Luminex Corporation, Austin, TX). To produce the highest resolution, the camera setting was at 1.0 μm per pixel of the charge-coupled device. In INSPIRE ^®^ FS data acquisition software, two brightfield channels were collected (channels 1 and 9), one FZ3 image (channel 2), one PI image (channel 5), one side scatter (SSC; channel 6), and one H33342 (channel 7), with a minimum of 10,000 spermatozoa collected. The following lasers and power settings were used: 405 nm (to excite H33342): 10 mW; 488 nm (to excite FZ3): 60 mW; 561 nm (to excite PI): 40 mW; and 785 nM SSC laser: 10 mW.

### 4.7. IBFC Data Analysis

The data were analyzed using IDEAS^®^ analysis software (AMNIS Luminex Corporation, Austin, TX), version 6.2. The gating approach used standard focus and single-cell gating calculations created by IDEAS software as previously described [8].

### 4.8. Fluorogenic Proteasomal Activity Assay

The protocol was adapted from [58] and [59]. Spermatozoa from the pre-rich and rich fractions were washed three times with the base buffer (50 mM Tris·HCl pH = 7.5, 40 mM KCl, 5 mM MgCl2). The washed sperm pellets were resuspended in the assay buffer (base buffer supplemented with 0.5 mM ATP disodium salt, 1 mM DTT, 0.05 mg/mL BSA, and 0.025 % digitonin). A total of 5 million spermatozoa in 100 µL of the assay buffer were loaded to each well of a 96-well plate. To study the effect of zinc ions on sperm proteasomal activity, ascending final concentrations of ZnCl_2_ were used for Zn^2+^: 15 µM, 0.5 mM, 1.5 mM, and 2.5 mM. The controls were run in parallel with 2.5 mM CaCl_2_, non-treated spermatozoa and proteasomally-inhibited spermatozoa with 100 µM epoxomicin. To monitor proteasomal activities, 100 µM of the following fluorogenic substrates were used: (i) Ac-GPLD-AMC to monitor caspase-like activity, (ii) Ac-RLR-AMC to monitor trypsin-like activity, and Suc-LLVY-AMC to monitor chymotrypsin-like activity.

The 96-well plates were read on by a 2104 EnVision Multilabel Plate Reader (PerkinElmer) using Open EnVision Manager software (ver. 1.13.3009.1401). The aliquots were incubated for 60 min at 37 °C with continuous agitation and subjected to excitation at 380 nm. Emission was recorded at 460 nm every 10 min. Five biological replicates were performed in total, with each containing four technical replicates.

### 4.9. Sperm Oviductal Glycan Binding Assay

Glycan-coated streptavidin-Sepharose High-Performance beads (GE Healthcare Bio-Sciences, Pittsburgh, PA, an average diameter of 34 µm) were used to test the ability of spermatozoa to detach from oviduct glycans (bi-SiaLN: biantennary 6-sialylated lactosamine oligosaccharide, suLe^X^: 3-O-sulfated Lewis X trisaccharide). To link the glycans to beads, approximately 60 µg of each glycan [60] covalently attached to a biotinylated polyacrylamide core were incubated with 20 µL of streptavidin-Sepharose beads for 90 min at room temperature. Each 30-kDa molecule of polyacrylamide had 20% glycan and 5% biotin, by molarity.

To prepare fibronectin-coated beads (positive-control for glycan coated beads), fibronectin (FN, Cat # 354008, Sigma-Aldrich, St. Louis, MO) was first biotinylated by incubating 45 µL of 10 mM biotin with 1 mL of a 1 mg/mL solution of FN, both in PBS. After incubation for 2 h at 5 °C, free biotin was removed using a desalting spin column. The biotinylated FN (60 µg) was incubated with 20 µL of streptavidin-Sepharose beads for 90 min at room temperature as above for biotinylated glycans.

Beads incubated with biotinylated FN and glycans were washed twice in NC-TALP (non-capacitating mTALP; NC-TALP; 2.1 mM CaCl_2_, 3.1 mM KCl, 1.5 mM MgCl_2_, 100 mM NaCl, 0.29 mM KH_2_PO_4_, 0.36% lactic acid, 0.6% polyvinyl alcohol, 1 mM pyruvic acid, 35 mM HEPES, pH 7.3, sterile filtered) and re-suspended in 100 µL of NC-TALP. Once the glycan-coupled beads were ready for use, a 50-µL droplet containing 1.5 million spermatozoa/mL was prepared to receive 1 µL of glycan-coated beads. Non-capacitated spermatozoa and beads were co-incubated for 45 min at 39 °C, 0% CO_2_. The number of spermatozoa bound per bead was counted before addition of any treatments. After that, an appropriate amount of Zn^2+^ was added (ZnCl_2_;15 µL, 0.5 mM, 1.5mM, 2.5 mM); as a control for divalent cation zinc, Ca^2+^ (CaCl_2_; 2.5 mM) was added. After incubation for 15 min at 39 °C, 0% CO_2_, progesterone (P_4_; 80 nM) was added to the droplets and incubated for 30 min at 39 °C, 0% CO_2_. For each treatment, 25 beads were randomly selected, and the total number of bound spermatozoa was enumerated. Spermatozoa that were self-agglutinated were not included in the counts. The experiment was documented using a Zeiss Axioskop and AxioCamHRc (Zeiss Microcopy, LLC, Thornwood, NY, USA).

### 4.10. Zymography Sample Preparation and Assay

Boar and bull spermatozoa were prepared for zymography as previously described [33], including the use of the trophoblast cell line known to express MMP2 and MMP9. Briefly, the detergent used to solubilize and extract inner acrosomal membrane (IAM) associated proteins was 1% non-ionic detergent Nonidet P-40 (NP-40) with sonication. The supernatant was separated from the pellet by centrifugation at 7000× *g* for 10 min at 4 °C and mixed with non-reducing sample buffer (200 mM Tris pH 6.8, 4 % SDS, 0.1 % bromophenol blue, 40 % glycerol) for analysis by zymography.

The samples were loaded onto 10 % SDS-polyacrylamide gels containing gelatin as previously described [33]. After electrophoresis, the enzymes were rinsed twice for 30 min, followed by an additional 1 h in 2.5% Triton X-100 (TrX), 5 mM CaCl_2_, and 50 mM Tris pH 7.5 in ddH_2_O at room temperature. They were then incubated overnight at 37 °C in solution void of TrX. MMP gelatinase activity was studied with and without the inclusion of 1.5 mM Zn^2+^ (with vehicle control already containing 5.0 mM Ca^2+^). The next day, the gels were stained with Coomassie stain and destained in 30% methanol, 10% glacial acetic acid, and 60% ddH2O for 2 hrs. Clear bands in the zymogram indicated enzymatic digestion of gelatin. The results shown are typical of two replicates.

### 4.11. Statistics

All results are presented as mean ± standard error of the mean unless otherwise noted. SAS 9.4 (SAS Institute, Inc, Cary, NC) GLM procedure and Duncan’s Multiple Range Test was used to analyze replicates. Bartlett and Leven tests found the sample sets to be homogenous. For proteasome activity assay, the data were analyzed as a randomized complete block split-plot in time design (as outlined by [61]) using the GLIMMIX procedure in SAS 9.4. The block is defined as an ejaculate. The main plot contains the effects of fraction (pre-rich vs. rich), treatment concentrations, and the interaction of fraction x concentration. The denominator of F for the main plot effects was block. The subplot contained the effects of time and all possible interactions of time with the main plot effects. The residual means square was used as the denominator for the subplot effects. Log transformation was performed when the data was not normal and/or unequal variances among means. The mean differences were determined using Fisher’s protected least significant difference (LSD).

## 5. Conclusions

Here, we show that the differences in the zinc signature based on ejaculatory sequence likely predestine the fertilizing cohort of spermatozoa, those that populate the oviductal reservoir (pre-sperm rich) and those that have already undergone early stages of sperm capacitation (rich and post-rich fractions). Others have previously shown pre-rich fraction spermatozoa to be more fertile than the rest of the ejaculate; however, biomarkers to explain the mechanistic differences between the vanguard group and the rest of the ejaculate have been lacking until now. Additionally, we show that in the presence of relatively high Zn^2+^, spermatozoa are incapable of release from oviductal glycans and the activation of sperm zona proteinase MMP2 and the 26S proteasome, likely necessitating the Zn^2+^ efflux observed during sperm capacitation. In the context of the fertilization-induced oocyte zinc spark and the ensuing oocyte-issued polyspermy-blocking zinc shield, the inhibitory effect of zinc on sperm-borne enzymes may contribute to the fast block of polyspermy at fertilization.

## Figures and Tables

**Table 1:** Boar ejaculate fraction endows sperm zinc signature subpopulations.

Fraction	Signature 1	Signature 2	Signature 3	Signature 4
Pre	93.5 ± 1.4% ^Aa^	3.7 ± 1.3% ^Ab^	2.0 ± 0.8% ^b^	0.8 ± 0.4% ^b^
Rich	2.8 ± 1.5% ^Ba^	91.5 ± 1.1% ^Bb^	3.9 ± 0.4% ^a^	1.8 ± 0.5% ^a^
Post	4.9 ± 2.1% ^Ba^	87.5 ± 1.8% ^Bb^	4.8 ± 1.8% ^c^	2.9 ± 0.6% ^c^
Whole	7.6 ± 4.4% ^Ba^	88.0 ± 2.4% ^Bb^	3.2 ± 1.5% ^c^	1.2 ± 0.6% ^a^

Data are presented as mean ± SEM. Values with different uppercase superscripts (A,B) indicate significant difference by fraction (*p*-value < 0.0001), and lowercase superscripts (a,b,c) indicate significant difference of zinc signatures within a fraction (*p*-value < 0.0001). Four biological replicates (*n* = 4) were analyzed with a total of 10,000 cells analyzed per sample.

## Supplementary Materials

Supplementary materials can be found at https://www.mdpi.com/1422-0067/21/6/2121/s1.

## Abbreviations

Zn^2+^	Zinc ions
ZP	Zona pellucida
AI	Artificial insemination
MMP2	Matrix metalloproteinase-2
FZ3	FluoZin ™-3
bi-SiaLN	biantennary 6-sialylated lactosamine 11-oligosaccharide
suLe^X^	3-O-sulfated Lewis X trisaccharide
DIC	differential interference contrast
P_4_	Progesterone
PI	Propidium iodide
IAM	Inner acrosomal membrane
PNA	Lectin peanut agglutinin
ZnR	Zn-sensing receptor
HVCN1	hydrogen voltage channel 1
H33342	Hoechst 33342
Epox	Epoxomicin
CLBL	clasto-Lactacystin β-Lactone
IBFC	Image-based flow cytometry
FN	Fibronectin
NP-40	Nonidet P-40
TrX	Triton X-100
SAS	Statistical Analysis Software
GLM	General Linear Model
GLIMMIX	Generalized linear mixed model
NIFA	National Institute of Food and Agriculture
USDA	U.S. Department of Agriculture
NIH	National Institute of Health
NSERC	Natural Sciences and Engineering Research Council
