# Supplementary material for: Sperm Cohort-Specific Zinc Signature Acquisition and Capacitation-Induced Zinc Flux Regulate Sperm-Oviduct and Sperm-Zona Pellucida Interactions
Source: Int J Mol Sci. 2020 Mar 19;21(6):2121. doi: 10.3390/ijms21062121 (PMC7139966; doi:10.3390/ijms21062121)
Supplement: Supplementary file 1 [file ijms-21-02121-s001.pdf]

Supplementary

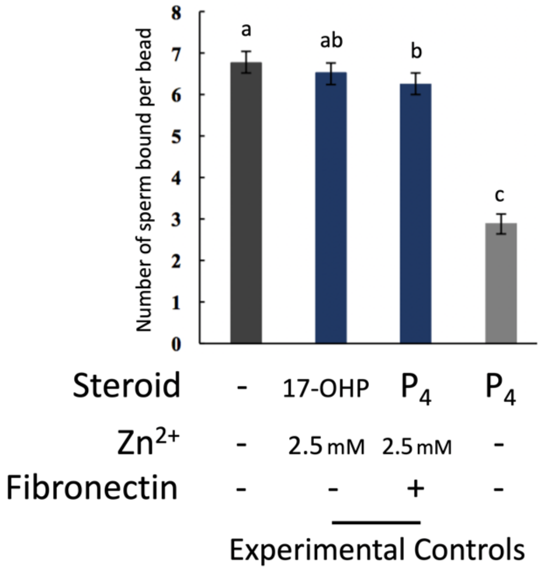

Figure S1. Controls for sperm release from glycans of the oviductal sperm reservoir. Values with different superscripts (a,b,c) indicate significant difference by treatment (p-value < 0.05).

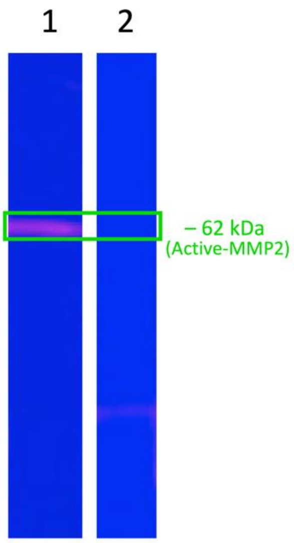

Figure S2. Zinc ions block the activity of the active form of human sperm-borne ZP proteinase MMP2. Clear band in the zymogram indicates enzymatic digestion of gelatin. Lane 1: vehicle block, lane 2: 1.5 mM $ZnCl_2$ block.

Table S1. Zinc inhibits P_4-induced sperm release from oviductal glycans.

Treatment	Number of Sperm Bound
Before Steroids	6.79 ± 1.29%^{A}
15 μM Zn^{2+} + P_4	4.32 ± 1.21%^{D}
0.5 mM Zn^{2+} + P_4	4.91 ± 1.30%^{C}
1.5 mM Zn^{2+} + P_4	5.95 ± 1.58%^{B}
2.5 mM Zn^{2+} + P_4	6.61 ± 1.29%^{A}
2.5 mM Zn^{2+} + 17-OHP	6.52 ± 1.29%^{AB}
2.5 mM Zn^{2+} + Fibronectin + P_4	6.25 ± 1.31%^{B}
2.5 mM Ca^{2+} + P_4	3.84 ± 1.28%^{E}
P_4	2.89 ± 1.18%^{F}

Table data for manuscript figure 3, presented as mean ± SD. Values with different uppercase superscripts (A,B,C,D,E,F) indicate significant difference by treatment (p-value < 0.05). Three biological replicates (n = 3) were analyzed.
